# Harvested populations are more variable only in more variable environments

**DOI:** 10.1002/ece3.2164

**Published:** 2016-05-24

**Authors:** Tom C. Cameron, Daniel O'Sullivan, Alan Reynolds, Joseph P. Hicks, Stuart B. Piertney, Tim G. Benton

**Affiliations:** ^1^ School of Biological SciencesUniversity of EssexColchesterCO43SQUK; ^2^ School of Biological SciencesUniversity of LeedsLeedsLS2 9JTUK; ^3^ Institute of Biological and Environmental SciencesUniversity of AberdeenAberdeenAB24 2TZUK

**Keywords:** Age‐truncation, density dependence, environment, harvesting, microcosm, mortality, population dynamics, predation, seasonality, stage‐structure, threshold, variability

## Abstract

The interaction between environmental variation and population dynamics is of major importance, particularly for managed and economically important species, and especially given contemporary changes in climate variability. Recent analyses of exploited animal populations contested whether exploitation or environmental variation has the greatest influence on the stability of population dynamics, with consequences for variation in yield and extinction risk. Theoretical studies however have shown that harvesting can increase or decrease population variability depending on environmental variation, and requested controlled empirical studies to test predictions. Here, we use an invertebrate model species in experimental microcosms to explore the interaction between selective harvesting and environmental variation in food availability in affecting the variability of stage‐structured animal populations over 20 generations. In a constant food environment, harvesting adults had negligible impact on population variability or population size, but in the variable food environments, harvesting adults increased population variability and reduced its size. The impact of harvesting on population variability differed between proportional and threshold harvesting, between randomly and periodically varying environments, and at different points of the time series. Our study suggests that predicting the responses to selective harvesting is sensitive to the demographic structures and processes that emerge in environments with different patterns of environmental variation.

## Introduction

A central challenge in ecology is to understand population responses to harvesting in a changing or variable environment (Hsieh et al. 2006; Anderson et al. 2008; Stenseth and Rouyer 2008; Fryxell et al. 2010; Bunnefeld et al. 2011; Shelton and Mangel 2011a; Rouyer et al. 2012). Such insight is a necessary prerequisite for the effective management and conservation of natural resources under long‐term environmental change. The developing consensus is that harvesting perturbs age‐ or stage‐structure, largely by causing an age‐truncation effect (ATE) through the removal of larger, older, or adult individuals. This reduces competition for resources between survivors and should therefore lead to greater synchrony in key demographic rates, such as somatic growth, development and maturation size. Relative to unharvested populations, the reduced competition in harvested populations will create more even age‐ and size‐ distributions among juveniles, and these new distributions cause different population responses to environmental perturbations. For example, if food becomes abundant, the more even juvenile age‐distribution can allow harvested populations to respond faster as there are more larger juveniles that can grow to adulthood faster and reproduce earlier relative to unharvested populations. Through increasing the likelihood of over‐compensatory responses, ATE can create an increase in population variability in variable environments (Anderson et al. 2008; Rouyer et al. 2010, 2012; Shelton and Mangel 2011a).

Recent and high profile studies addressing the interaction between harvesting and population variability have taken one of two approaches (Hsieh et al. 2006; Ottersen et al. 2006; Anderson et al. 2008; Stenseth and Rouyer 2008; Rouyer et al. 2010, 2012; Shelton and Mangel 2011a, 2011b; Sugihara et al. 2011). They either consider comparable data from fish species that vary in their harvesting pressures, or focus on detailed time series of well‐studied fish stocks and look at their dynamics through periods of low and high adult fishing mortality or climate variation. The conclusions drawn from both approaches arise from statistical analyses to separate the effects of mortality and environmental noise on population variability. The majority of studies, despite their different approaches, come to similar conclusions about marine fisheries, namely that harvesting changes the ratio of large or old individuals to small or younger individuals and this leads to stronger nonlinear population dynamic responses to environmental variation (Anderson et al. 2008; Shelton and Mangel 2011a; Rouyer et al. 2012). While contentions remain (Shelton and Mangel 2011b; Sugihara et al. 2011), there is increasing evidence to support the view that the driver of over‐ and under‐compensatory responses of harvested populations to environmental variation is that harvesting changes the population's response to environmental changes through alteration in demographic structure and abundance (Cameron and Benton 2004; de Roos et al. 2007). These impacts can arise both directly, by mortality changing numbers, and indirectly, by mortality changing the competitive environment leading to changes in the survivors’ intake rates, growth, maturity and fecundity. We would highlight the mechanistic similarity between harvesting and its impacts on fish population dynamics and studies considering the interaction between harvesting and environmental noise on population variance in terrestrial vertebrates (Bunnefeld et al. 2009, 2011; Chapman et al. 2009), and earlier studies with invertebrate model organisms in microcosm (e.g., Benton et al. 2002, 2004; Cameron and Benton 2004).

Despite the increasing evidence of the complexity of animal population dynamics in variable environments, much of its underpinning theory has typically been derived by exploring the consequences of adding stochasticity, representing environmental variation, directly to terms in otherwise unstructured deterministic models (e.g. Shelton and Mangel (2011a), and see discussion in Ranta et al. (2000)), or correlating animal abundance with temporal variation in abiotic variables such as temperature or the North Atlantic Oscillation (Grenfell et al. 1998). This has led to many different predictions where increasing environmental variation can be shown to either increase or decrease population variability in response to harvesting. A recent theoretical study flagged the need for experimental studies that would help determine under what types of environmental variation harvesting compounds or ameliorates population variability in structured populations living in dynamic environments (Wikstrom et al. 2012).

Experimental manipulation of populations in microcosm is a powerful tool to provide very detailed data that can inform a mechanistic understanding of how populations respond to perturbations, variable environments, and harvesting regime (Cameron and Benton 2004; Fryxell et al. 2005; Schroder et al. 2009; Nilsson et al. 2010; Strevens and Bonsall 2011). Specifically because field tests are rare and difficult (though possible see Ohlberger et al. (2011)), experimental microcosm approaches provide a way to explore the broad responses of live organisms to management‐like interventions and ultimately influence field practitioners (Benton et al. 2007). Experimental ecology & evolution, usually undertaken on invertebrates, often capture the complex population and community dynamics that are also seen in higher taxonomic order animals such as delayed feedback or generation cycles (Mittelbach and Persson 1998; Bjornstad et al. 1999; Wearing et al. 2004; Cameron et al. 2007), positive density dependent responses to mortality (Benton et al. 2004; Butler et al. 2009; Schroder et al. 2009) and eco‐evolutionary dynamics in response to environmental change and size‐structured or trophy harvesting (Cameron et al. 2013, 2014; Smallegange and Deere 2014).

Here, we study the interaction between environmental variation and selective harvesting on population parameters in a well‐studied model system, the soil mite *Sancassania berlesei*. We conduct this study with weekly census information over 102 weeks (equivalent to 20 generations). We maintain populations in dynamic environments characterized with different regimes of daily food supply (constant, variable, or periodic) while keeping the total supply constant. Populations are harvested or unharvested, with the former involving weekly removal of a 40% of adult numbers estimated from a simulation model (Benton 2012). The harvesting regimes are either proportional (harvesting a fixed 40% of the nonharvested population size) or threshold (i.e., removing all adults above 60% of the nonharvested population size). Previously, using this time series, we have shown that the life history of wild soil mites evolves in response to the average conditions in a laboratory environment and this determines the persistence of these populations by changing the population trajectory from decreasing to an increasing or stable equilibrium (Cameron et al. 2013). We have also reported that the environmental variability present in these experiments has much less of an influence on the evolution of mean values of life history traits than the average laboratory conditions, but there was a positive relationship between increased environmental variation and increased evolved age at maturity (i.e., generation time) (Cameron et al. 2014).

Here, we focus specifically on addressing how variation in population abundance is influenced by the interaction between selective harvesting and environmental variation in food supply. For clarity we also report concurrent changes in mean population sizes across all treatments, some of which have been summarized previously (Cameron et al. 2013, 2014). We have a detailed understanding of demographic mechanisms underpinning density dependence in this system and using this we can investigate: (1) population dynamics data arising via frequent censuses (Benton et al. 2001b, 2004, 2006; Beckerman et al. 2002; Benton and Beckerman 2005; Benton 2012); (2) where we have manipulated the environmental variation (Benton et al. 2002; Benton and Beckerman 2005); (3) and where we have also imposed harvesting near the maximum sustainable yield, proportionally (density‐independent) and at a threshold (density‐dependent) (Benton et al. 2004; Cameron and Benton 2004). Specifically, we ask whether different harvest strategies create populations that are more or less stable and whether harvested populations show greater responses to environmental variation than nonharvested controls.

## Methods

### Experimental treatments and population census

Wild soil mites were collected from Chicken manure and compost from Aberdeenshire and horse manure and garden soil from West Yorkshire, and reared using standard techniques described elsewhere (Benton et al. 2001a).

Fifty to 100 mites from each location were reared for one generation (4–6 weeks) in excess food before being mixed together for a further generation. Forty‐two uniform small glass tubes (soda glass, 25 mmØ, 50 mm tall, filter paper seal and press on lid) half‐filled with experimental grade calcium sulfate were each inoculated with 150 males, 150 females, and 1000 juveniles of unknown sex at the start of the experiment (day 1 of week 1). Our main results presented here are from near the end of the experiment beyond any possible ecological transients.

Tubes were assigned to constant (12 replicates), randomly variable (18 replicates), or a 28 day periodically variable (12 replicates) food provision treatment. All treatments received the same average daily food input of 2 × 0.0015 g balls of dried active yeast, over the course of the experiment. In the constant treatment, every tube received two balls/day. In the random treatment, every tube received between zero to 13 balls/day, but with a distribution based on a negative binomial, with mean of 2 and dispersion parameter of 0.5 over a 56 day period. The periodic treatment mimicked a seasonal environment with food provided in the following repeating pattern: 9 days of no food, 3 days one ball yeast, 2 days three, 9 days four, 3 days three, and the final 2 days in the cycle one ball. Over these 4 week periods, the variance in total food delivery per week, expressed as a coefficient of variation (CV) averaged zero, 0.36 and 0.86 for constant, random, and periodic food respectively. Each tube received at least two drops of distilled water/day to maintain humidity. Replicate tubes from each environmental variation treatment were randomly assigned to different experimental harvesting treatments; unharvested or proportional harvested (where 40% of adults were removed from the population per week) or threshold harvested (where all adult individuals above 60% of the long‐term predicted adult population size were removed = above 173 adults; *n* = 6 replicate tubes per harvesting treatment). The threshold harvest treatment therefore experiences weeks where densities are too low and harvesting does not occur, and over the later part of the time series that we examine in greater detail this resulted in an average weekly harvest rate of adults of 30.1 ± 5.1%. The threshold harvested treatment was only conducted in the randomly variable environment treatment. Pilot experiments and simulations of an individual based model described in (Benton 2012), provided predictions that adult harvest rates much above 40%/week would not be sustainable in the long‐term.

Population tubes were counted in the morning on the same day each week under a Nikon SMZ1500 stereomicroscope with attached cool LED ring light (Nikon UK Limited, Kingston Upon Thames, UK). When harvesting was required the required number of individuals was removed using a fine brush. Harvesting began on week 13 and ended in week 83. The mite generation time is approximately 30–37 days under relatively constant conditions (Ozgul et al. 2012). To measure the size distribution of juveniles (all stages combined) and adults, photographs of the entire area of each replicate tube were taken using a Nikon Digital Sight 5.0 megapixel camera (DS‐5M) attached to a Nikon SMZ1500 stereomicroscope at ×1.5. These photographs were captured during week 60 near the end of the time series when the populations had been exposed to environmental variation and harvesting for 47 weeks. Longest length measurements of all individual mites that were not obscured in the image were recorded using Nikon software (Elements‐D).

### Analysis

From the 102 week time series of 42 populations (Figure S1) we first estimated the temporal patterns of change in coefficients of variation (CVs) of adult abundance. The CV is used as a measure of population variance relative to the mean (which changes with time and treatment) and calculated in 5‐week centered moving windows (using function “rollapply” in package “zoo” in R3.0.2).The CV for a week *i* in the times series is calculated from the adult abundance over weeks *i*−2 to *i* + 2 which is approximately equal to the generation time (Ozgul et al. 2012).

To describe and visually compare between trends in the CV of adult population sizes per environment × harvest treatment, we fitted general additive models (GAMS) across the CV of population size of each replicate population tube as a function time (weeks), and constrained the intercepts to be the average CV of population size at the beginning of the experiment. The best GAMs were chosen by selecting the model(s) with the lowest AIC score between those with df of 1–10, 15, 20. The best model is then used to plot the smoothed mean CV and confidence interval over the course of the experiment to visually compare differences between treatments on population variability. Additional analyses over 10‐ or 20‐weeks centered moving windows of CV of adult population size can be found in the Supporting online information, but remain qualitatively similar. Plots of trends in mean population sizes in response to environment and harvesting have been presented in Cameron et al. (2013, 2014), but can also be found in the Supporting information.

**Figure 1:**
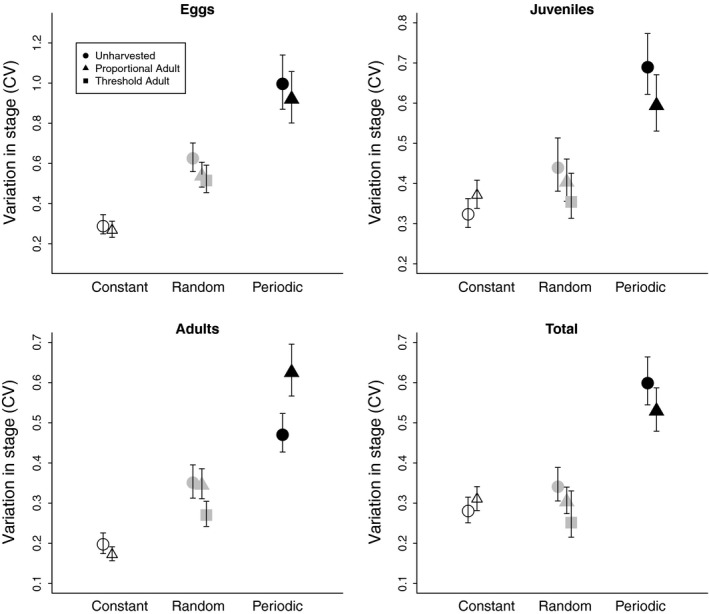
Bootstrap resampled and stratified coefficient of variation of population size (mean and 95% confidence intervals) for weeks 60–80 (detrended) for each stage in the three environmental variation and associated stage‐selective harvest treatments. Environmental variation increases from zero (coefficient of variation), to 0.36 to 0.86 for constant, randomly variable and periodic in weekly food treatments. Symbols refer to different harvesting treatments. Error bars are bias corrected and adjusted 95% confidence intervals of the mean and those that do not overlap the mean of a comparable treatment can be considered statistically different at α = 0.05. Scales differ between panels.

**Figure 2:**
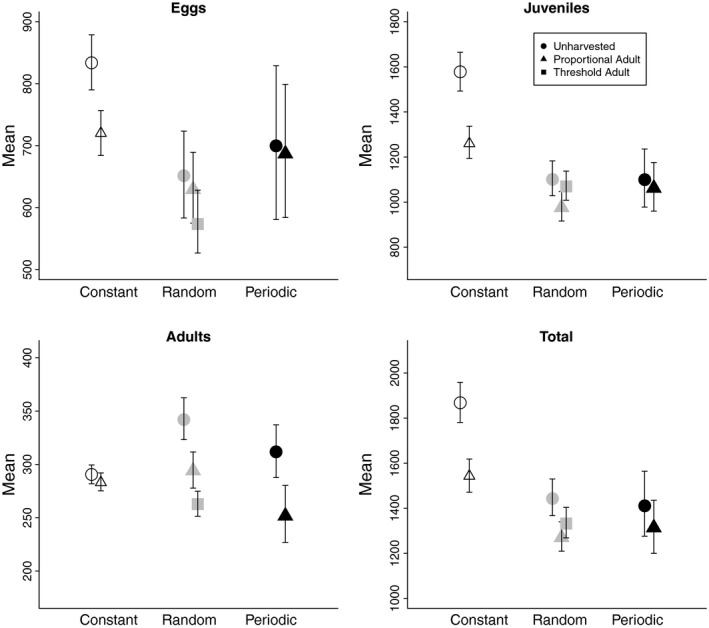
Bootstrap resampled and stratified mean population size (mean and 95% confidence intervals) for weeks 60–80 (detrended) for each stage in the three environmental variation and associated stage‐selective harvest treatments. Environmental variation over 4 week period increases from zero (coefficient of variation), to 0.36 to 0.86 for constant, randomly variable and periodically supplied food treatments. Symbols refer to different harvesting treatments. Error bars are bias corrected and adjusted 95% confidence intervals of the mean and those that do not overlap the mean of a comparable treatment can be considered statistically different at α = 0.05. Scales differ between panels.

For a detailed assessment of the relative impacts of the harvesting and environment treatments on population variation (CV) and mean abundance, we chose a 20‐week time series (weeks 60–80, 3–4 generations) toward the end of the experiment such that the response was assessed after approximately 12 generations of exposure to environmental variation and harvesting, and assume this window is (1) small enough for the time series to be approximately stationary, (2) following the erosion of initial ecological transients and thus with approximately stable age/size distributions, and (3) following the most intense period of natural selection, and thus evolutionary change (Cameron et al. 2013). We will refer to the GAM predicted plots of trends that describe where any variation in the length or period of this 20‐week time series may have influenced our conclusions from selecting weeks 60–80. The population and stage means and CVs of abundance for each treatment combination were calculated using stratified bootstrap resampling of the time series data across all six replicates to give robust estimates that account for repeated measures (Benton et al. 2002; Cameron and Benton 2004). To compare the CVs across different treatments that differed in their rate of change in mean population size, the time series were first detrended by subtracting a locally weighted polynomial regression model of the mean trend (i.e., lowess). The bootstrap mean and 95% confidence intervals of population CV or mean abundance are presented in figures and percentage differences between these mean treatment values and the controls are provided in the text. Statistical significance was estimated with linear models, chosen based on a prior analysis (e.g., Benton et al. 2002), to consider the effects of type of environmental variance (factor: constant, randomly variable, or periodic) on the population statistics within each harvest treatment. Statistical significance of the effects of harvesting, within each life history stage and environmental variation treatment, were derived from the bootstrapped 95% confidence intervals as also presented in Figures 1 and 2, that is a 2 × standard error distribution/*t*‐test. Where differences were not significant they are referred to as “ns” in the main text. A summary of the effect sizes of environmental variance and harvesting, as provided throughout the results text, can be found in Tables S1 and S2.

To test for differences in the size structure of populations from different environments or harvest treatments the mean and confidence interval of the size probability density function was calculated for both the constant and periodic environment populations using the R 3.0.2, package “stats”, function “density” (*n* = 8192 measurements).

Supporting information includes details of some statistical analyses reported below in Tables S1 and S2.

## Results

### Effects of environmental variation and harvesting on population variability

Environmental variation has a large and statistically significant effect on the CV of total population and stage‐specific dynamics during weeks 60–80 (Fig. 1, Table 1). Periodic environmental variation increases total population and stage variation more than random environmental variation, for example the CV of adult number from unharvested populations are 0.2, 0.34, and 0.46 in constant, random, and periodic environments respectively (anova Unharvested Adults~Environmental Variation, *F*_1,15,_ = 32.77, *P* < 0.001).

**Table 1:** Coefficient of variation of population size as a function of environmental variation (var: 0 = constant, 1 = random, 2 = periodic) for each stage and the total population

Stage	Unharvested	Adult harvested
Eggs	CV = −1.45 (±0.09) + 0.63 (±0.07) var	CV = −1.52 (±0.06) + 0.67 (±0.05) var
	*R* ^2^ = 0.83, *F*_1,16_ = 75.94, *P *=* *1.798e^−7^	*R* ^2^ = 0.91, *F*_1,16_ = 165.0, *P *=* *7.61e^−10^
Juveniles	CV = −1.48 (±0.09) + 0.26 (±0.07) var	CV = −1.47 (±0.11) + 0.26 (±0.08) var
	*R* ^2^ = 0.49, *F*_1,16_ = 15.43, *P *=* *0.0012	*R* ^2^ = 0.38, *F*_1,16_ = 9.738, *P *=* *0.006
Adults	CV = −1.90 (±0.09) + 0.41 (±0.07) var	CV = −1.90 (±0.06) + 0.49 (±0.05) var
	*R* ^2^ = 0.65, *F*_1,16_ = 30.88, *P *=* *4.33e^−5^	*R* ^2^ = 0.88, *F*_1,16_ = 113.4, *P *=* *1.14e^−8^
Total	CV = −1.67 (±0.09) + 0.27 (±0.07) var	CV = −1.68 (±0.12) + 0.31 (±0.09) var
	*R* ^2^ = 0.49, *F*_1,16_ = 15.53, *P *=* *0.00117	*R* ^2^ = 0.40, *F*_1,16_ = 10.51, *P *=* *0.005

Harvesting adults has little effect on the variation in abundance of any life history stage in a constant environment (Fig. 1, adult harvest eggs 5.8% less ns, juvs 15.6% more, adults 15% less ns). Harvesting adults as a fixed proportion in randomly variable environments has no statistically significant effect on variation in adult numbers (both have a CV of approximately 0.35 see Fig. 1 and “Discussion”), but reductions in variation of other stages eggs 14% less, juveniles 8% less ns). Harvesting adults in a periodic environment leads to large and significant increases in variation in adult abundance (32.9% more variation in adults than unharvested control) and concurrent reduction in variation in other stages (eggs 7.7% less, juveniles 13.8% less, Fig. 1).

### Effects of environmental variation and harvesting on population size

During weeks 60–80, in the unharvested populations, the total population size is reduced in random environments relative to the constant environment (Fig. 2, Average_random_ 22.2% less, *t*_1,11_ = −2.9, *P* < 0.02; Average_periodic_ 24.4% less, *t*_1,11_ = −2.7, *P* < 0.02, Table 2). This reduction in total population size is primarily due to the reduction in numbers of the numerically dominant eggs and juveniles, whereas adult population sizes are larger in random environments relative to the constant control (Fig. 2, adult numbers Average_random_ 17.2% more, *t*_1,11_ = 2.2, *P* < 0.05; Average_periodic_ 7.26% more, *t*_1,11_ = 0.9, ns).

**Table 2:** Ln population size as a function of environmental variation (var: 0 = constant, 1 = random, 2 = periodic) for each stage and the total population

Stage	Unharvested	Adult harvested
Eggs	ln(Pop) = 6.326 (±0.045) − 0.129 (±0.035) var	ln(Pop) = 6.279 (±0.036) − 0.126 (±0.028) var
	*R* ^*2*^ = 0.46, *F*_1,16_ = 13.72, *P *=* *0.0019	*R* ^2^ = 0.56, *F*_1,16_ = 20.62, *P *=* *0.00033
Juveniles	ln(Pop) = 7.043 (±0.072) − 0.302 (±0.056) var	ln(Pop) = 6.886 (±0.038) − 0.208 (±0.030) var
	*R* ^2^ = 0.65, *F*_1,16_ = 29.18, *P *=* *5.88e^−5^	*R* ^2^ = 0.75, *F*_1,16_ = 48.63, *P *=* *3.14e^−6^
Adults	ln(Pop) = 5.577 (±0.035) − 0.031 (±0.027) var	ln(Pop) = 5.607 (±0.048) − 0.050 (±0.037) var
	*R* ^2^ = 0.07, *F*_1,16_ = 1.29, *P *=* *0.27 NS	*R* ^2^ = 0.10, *F*_1,16_ = 1.833, *P *=* *0.194 NS
Total	ln(Pop) = 7.256 (±0.051) − 0.224 (±0.039) var	ln(Pop) = 7.136 (±0.031) − 0.172 (±0.024) var
	*R* ^2^ = 0.67, *F*_1,16_ = 31.82, *P* = 3.68e^−5^	*R* ^2^ = 0.76, *F*_1,16_ = 50.57, *P* = 2.47e^−6^

NS* = *nonsignificant.

The effects of harvesting on population and stage densities are dependent on the environment (Fig. 2). Harvesting adults reduces the total population size in a constant environment, due to a larger and statistically significant reduction of eggs and juveniles than adults (Adult harvest eggs 13.6% less, juveniles 20.1 less, adults 2.6% less ns). Harvesting adults in a randomly variable environment has no effect on eggs (−3.4% ns), but significant reduction in juveniles and adult numbers (juveniles 10.1% less, adults 14% less). This results in a significant reduction in total population size (12.3% less). Harvesting adults in a periodic environment has no effect on egg or juvenile numbers relative to controls, but significantly reduces adult abundance (Adults 19.3% less). This does not result in a statistically significant net reduction in total population size.

### How does variability in population size change across the time series?

**Figure 3:**
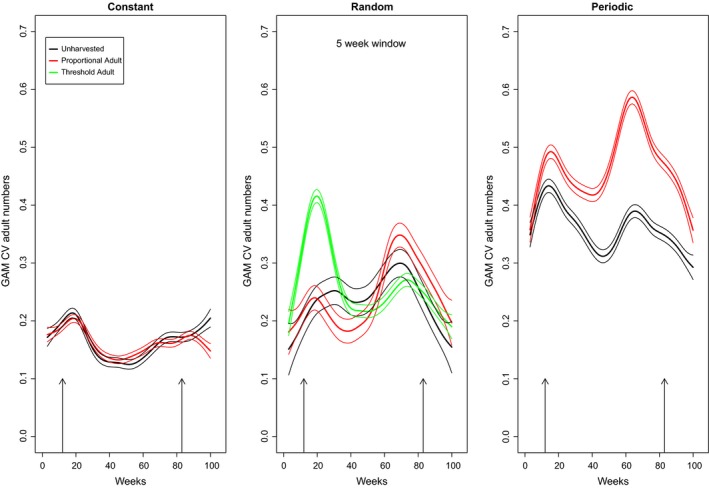
Each plot shows the fitted time series of mean ± 1 SE coefficient of variation (CV) of adult stage abundance as predicted from a General Additive Model (GAM) fit to the CV of adult abundance over a centered moving 5 week window. Time series are shown for unharvested, proportional and threshold harvest populations in constant (left column), randomly variable (middle) and periodic food supply (right) environments. Degrees of freedom (df) for the GAMs were chosen through model simplification, and determining the minimum df that could best represent all CV time series within 5 week centered moving windows (i.e., 6 df). Arrows show start and end of harvesting.

The CV of adult population size changes over time in the constant environment, regardless of harvest treatment, such that throughout most of the experiment there were no significant differences in adult number CV between control and adult harvested populations (Fig. 3). Similarly, the CV in adult numbers varied through time in random environments. Changes in the estimated variation in all environments near the end of these time series, beyond week 80, are influenced by the end points and the short nature of the postharvest time series. The key point however is whether there are changes in the relative differences in the responses of populations to harvesting across the time series and in particular, do they ever differ from the week 60–80 period when we looked at the changes in population summary statistics in detail.

Proportional harvesting of adults in randomly variable environments initially reduced the CV of adult numbers relative to the control, but this is reversed nearer the end of the experiment (Figs. 1, 3). The onset of harvesting leads to a large ecological transient of reduced variation of adult numbers during the initial decline in population size. Harvesting adults later in the time series as the population recovers – which we now know to be driven by evolution of the life history – results in variation in adult numbers increasing to larger than that in unharvested populations. A similar initial and large transient increasing CV in adult numbers in response to adult harvesting was seen under threshold harvests in randomly variable environments. This CV of adult numbers with a threshold harvest increases later in the time series, but always remains lower than in proportional harvesting (Fig. 3). Proportional harvesting of adults in periodic environments increased variation in adult population abundance relative to the control throughout the entire experiment (Fig. 3).

### Impact of different harvesting strategies on dynamics

We compared proportional harvest of 40% of adults/week and threshold harvesting of all adults above 60% of the predicted long‐term mean of an unexploited population (i.e., carrying capacity) under random environmental variation in weeks 60–80. Variation in adult population size and the mean population size are reduced more by threshold harvesting relative to both the unharvested control and proportional harvesting of adults (CV of adult population size: threshold harvest 22.9% less, proportional harvest 1.8% less than control ns, Fig. 1; average adult population size: threshold harvest 23.1% less, proportional harvest 14% less than control, Fig. 2). Converting this into estimated yields indicates that the different harvesting strategies significantly affect both the expected yield (*t*_1,11_ = 4.94, *P* < 0.01) and its variability: for threshold harvesting, the yield ± 2 SE is estimated as 6469 ± 771 adults, with a variation in yield over time, estimated by the CV, being 0.98 ± 0.12. Conversely, for proportional harvesting, the yield is estimated as 8366 ± 559 adults with a much lower CV at 0.32 ± 0.01.

### Impact of harvesting on the distribution of body sizes

**Figure 4:**
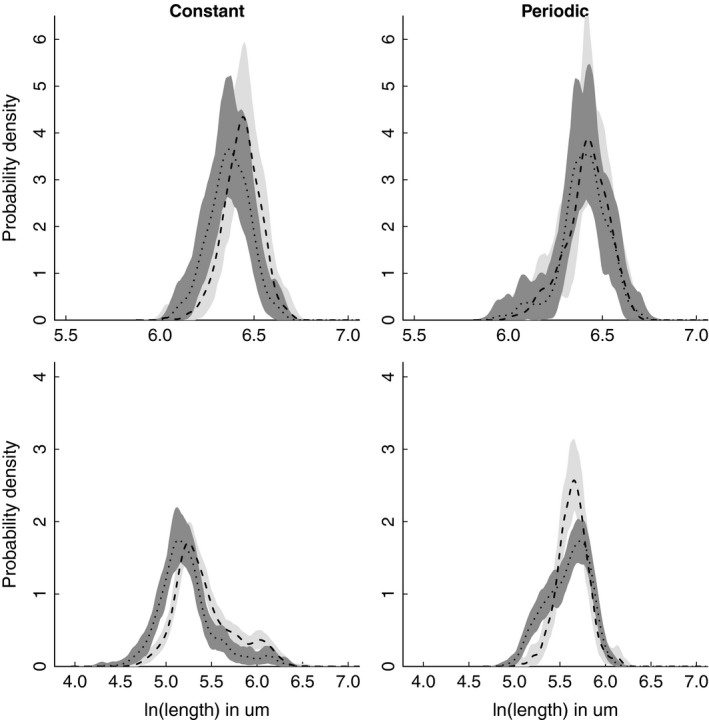
The probability density distributions of individual body lengths (log, μm) of adult female (upper panels) and juvenile mites (lower panels) from constant (left panels) or periodic (right panels) food supply populations which are unharvested (light gray/dashed line) or adult harvested (dark gray/dotted line). Harvest mortality was proportional with a rate of 40%/week for both targeted stages. Shaded error polygons show the 95% bootstrapped confidence envelope (*R* = 10,000) around the mean.

The size distributions of adult females from constant or periodic environments in weeks 60–80 are similar. Under constant food, selective harvesting of adults truncates the adult female and juvenile size distribution (Fig. 4). But the overall effect is small (constant environment: unharvested population female body length 625 *μ*m ± 3.25 SE vs. adult harvested female 586 ± 4.4 SE = 7% reduction). In periodic environments harvesting has almost no effect on the adult size distribution over this period. Adult harvesting however, significantly shifts the distribution of juvenile sizes in periodic environments (Fig. 4; periodic environment: unharvested population juvenile body length 285 μm ± 1.71 SE, adult harvested 273 *μ*m ± 1.81 SE).

## Discussion

Harvesting adults increased stage‐specific and total population variability in variable environments, but not in constant environments. Where mite populations were exposed to environmental variation for many generations it is clear that harvesting of adults leads to increasing variability of adult numbers. The magnitude of this effect increased over time, in both randomly and periodically variable environments, and could be a result of the previously identified life history evolution occurring over the course of the experiment (Cameron et al. 2013, 2014). Whether this is caused by evolution of more plastic phenotypes, greater genotype diversity in variable environments (e.g., disruptive selection) or both remains to be tested. We found no significant effect of harvesting on the relative population variability (CV) when the environment is constant.

The most parsimonious explanation why adult harvesting mortality increases adult population variance only in the presence of environmental variance is that age/size truncated populations are more likely to respond in an over compensatory way to mismatches between their age/stage/size structure and the immediate carrying capacity of the environment (Cameron and Benton 2004; Rouyer et al. 2012), due to an over representation of younger, more fecund, adults. Similarly age truncated populations are most likely to suffer highest mortality from short‐term negative environmental changes due to the investment in adult survival strategies in most high fecundity species (e.g., age/size truncated populations have relatively more vulnerable young/small individuals). Therefore, our results empirically support a variety of similar conclusions in other studies examining the cause of increased variability of wild populations – that both the effects of harvest on demography and environmental variation have a role (Hsieh et al. 2006; Anderson et al. 2008; Rouyer et al. 2010, 2011, 2012; Shelton and Mangel 2011a, 2011b; Sugihara et al. 2011).

We also found that harvesting adults does not always have the same effect on the variance of different ontogenetic stages. For example, in the periodic food environments harvesting adults leads to increased variance in adult and reduced variance in juvenile numbers, because removing adults during the periods when there is no food available reduces adult numbers with limited compensatory juvenile growth or maturation. In contrast, removing adults when food is available results in increased adult numbers via over‐compensatory adult recruitment without reduction in juvenile numbers (as juvenile recruitment and survival are high). A plot of long‐term average of adult and juvenile densities per replicate population tube misses this pattern (Figure S2). Likewise harvesting adults in a constant environment reduces the overall variance across all stages by decreasing variance in egg and juvenile but not adult numbers – because total population CV remained constant while total population size declined.

### What are the relative roles of harvest‐induced age or size‐truncation and environmental variation on population variability?

In our experiment, adult survival rates declined with age by approximately 6%/day under adult harvesting (i.e., calculated as the average change in daily survival over 7 days from emergence to the first harvest event, if harvesting was continuous). While no age‐specific data are available, the size frequency distributions of adult females from adult harvested constant environment populations are left skewed, caused by a decrease in the number of individuals over 650 *μ*m and increase in those under 550 *μ*m in adult harvested populations. In periodic populations, we see a smaller average size across the whole population. Overall, we have larger numbers of smaller adult females and juveniles; we would strongly argue that the average age has also reduced. However, this age truncation alone did not lead to increased population variation when environmental variation was minimized in our constant environment treatment.

Only when environmental variation and harvesting were combined did we see strong evidence of magnified fluctuations in abundance as in studies of commercial fisheries or harvested game birds (Anderson et al. 2008; Chapman et al. 2009; Rouyer et al. 2010, 2011, 2012; Bunnefeld et al. 2011; Shelton and Mangel 2011a). Thus, age‐truncation (or size‐truncation) from stage and/or size‐selective mortality interacts with environmental variation to increase population variability. This supports our earlier point that mismatches between population structures and the environmental state at any one time, leads to harvesting magnifying population variability – age/size‐truncation effects on their own are not enough. A second consideration of ATE is the effect that reducing females’ average age has on egg production and survival (Plaistow et al. 2007; Cameron et al. 2014). We find that harvesting adults can often reduce total population size through its influence on eggs and juveniles, as observed in constant and randomly variable environments. Female age is an important determinant of fecundity in soil mites (Plaistow et al. 2007), as in many species including fishes (Harris et al. 2007; Kaeding and Koel 2011; Targonska et al. 2012; Aliniya et al. 2013; Valentin et al. 2015), and changes in average female age can have lasting effects on juvenile survival, population variability, and size (O'Farrell and Botsford 2006; Plaistow and Benton 2009; Hsieh et al. 2010; Hixon et al. 2014; Shelton et al. 2015).

### Empirical testing of current theory on harvesting and environmental variation

Not all theory on harvested populations concludes that harvesting destabilizes dynamics. A recent study of variance in age‐structured model populations with stochastic recruitment differentiates between a constant and proportional harvest (Wikstrom et al. 2012). Wikstrom et al. found that under constant harvest rates removal of adults leads to higher variance due to an ATE whereas under a proportional harvest, as we used in our experiment, harvesting can increase or decrease population variance. Specifically proportional harvesting of adults can dampen population variance through reducing juvenile production (i.e., Allee effect), and through reductions in over‐compensatory responses in life histories close to the unstable dynamics boundaries. In our constant environment treatment proportional harvesting reduces the juvenile to adult ratio but not via an Allee effect, instead it is likely to be a maturation rates increasing in response to adult mortality (Cameron and Benton 2004). Higher maturation results in younger females less able to respond to any available food, and there will be less excess food in a constant environment (Benton et al. 2005; Plaistow et al. 2006). Proportional adult harvest leads to stabilizing effects in only a small percentage of cases in the Wikstrom et al. model, and is instead most often destabilizing in populations in autocorrelated environments as we found here. This occurs in tandem with an increase in the juvenile to adult ratio (i.e., age truncation) and suggests that harvest induced ATEs on population variability are more likely in populations living in positively autocorrelated environments (Wikstrom et al. 2012). However, we recommend caution in concluding that this result, and ours, corroborates fully the analysis of wild harvested populations.

A series of analytical studies that support the hypothesis that harvesting causes population variability in the wild, used a census of very young larval fish stages as their index of population abundance (CalCOFI Ichthyoplankton database) (Hsieh et al. 2006; Anderson et al. 2008). A case is then made that the biomass of larval fish is a “well known proxy for current (spawning) adult biomass” (Anderson et al. 2008; CEFAS, 2012). In our data we found no statistically significant evidence that harvesting adult mites in variable environments increases egg or juvenile stage variation. Neonate mortality is high in the mite model system as it is in marine fishes, up to 90%, and therefore egg counts are a best indicator of the abundance of the first mite juvenile stage (Benton et al. 2002; Cameron and Benton 2004; Ozgul et al. 2012). Where harvesting adults in a constant environment led to increased juvenile stage variability, this did not lead to significant increases in adult stage variance. Therefore, we would urge caution in the interpretation of studies on only egg or juvenile stage abundances in harvested populations for two reasons. Firstly compensatory changes in survival, growth, recruitment, or species interactions in later stages are likely to mask or modify the abundance and variability of any later stages, whether in model, microcosm, or wild populations (Bystrom et al. 1998; Persson et al. 2000; Ratikainen et al. 2008; Wikstrom et al. 2012). Secondly, despite its wide use due to no other fisheries independent information being available, a number of biases have been identified and cautions issued on the links between ichthyoplankton survey data and adult biomass in a variety of harvested species (Bernal et al. 2012; Kraus et al. 2012).

### Effects of harvesting and environmental variation on population size

We have shown previously that both increased environmental variation and harvesting can, separately, reduce population size (Benton et al. 2002; Cameron and Benton 2004; Cameron et al. 2013, 2014). Here, we extend these studies to show that the effects of harvesting on stage and total population size declined with increasing environmental variance, and that the stage‐specific effects of harvesting adults differ markedly between constant and variable environments. In a constant environment harvesting adults significantly reduces egg and juveniles stages but not adults. The alternate is true in variable environments. We suggest that environmental variance obscures or dampens harvest effects on total population size as, when food and population sizes are low; the smaller yield under a proportional adult harvest is easily compensated for via increased reproduction or juvenile survival before the next census or harvest. When food is plentiful any harvest rate is easily compensated for through increased juvenile growth, survival, and maturation due to the mismatch between the reduced postharvest density and the increased resources. However, under constant conditions resource competition is invariably high (Cameron and Benton 2004). Therefore, while competitively dominant larger juveniles can obtain the extra food following a harvest and quickly mature, smaller juveniles and females are not best placed to respond to increasing food availability and increase growth, maturation, and fecundity rates.

### Examining the effects of different harvesting regimes

Density‐dependent adult mortality from threshold harvesting, also referred to as fixed escapement (Fryxell et al. 2005), is thought to be a more precautionary harvest method as the harvest effort is proportional to the long‐term average of the sustainable yield. In this way it is thought to reduce the risk of extinction by reducing variability and preventing low population sizes through reducing harvest when population size is reduced (Lande et al. 1997; Fryxell et al. 2005). In our study, a threshold method restricted to harvest all adults above 60% of the long‐term population mean reduced long‐term averaged harvest effort, through the absence of harvesting in multiple weeks, resulting in an average harvest of close to 30%; 10% lower than the proportional harvest target rate of 40%. However, threshold harvesting of adults in randomly variable environments reduced adult densities by approximately 23%, compared to a 15% reduction by density independent proportional harvesting of adults. The increased reduction of adult population size via threshold management could occur via harvesting rate being too high, overharvesting, or too low to promote positive density‐dependent feedback from the mortality rate on adult recruitment (Schroeder et al. 2014).

While we have previously shown that soil mites exhibit positive effects of mortality (PEM), and the specific response is dependent on environmental variation (Cameron and Benton 2004), a 40% harvest mortality per week is likely to be too high to expect over‐ compensatory responses. Instead we suggest that as average harvest rates in the first 10 weeks across the variable threshold treatment populations were 45%, with several harvesting events in each replicate population being equivalent to 50–70% of the weekly mean adult population size, the threshold harvest method overharvested early on in the experiment from which the populations never recovered. This is consistent with the time series where adult population sizes in this treatment were reduced early on in the experiment. As originally predicted, the threshold harvest method significantly reduced variation in adult abundance over the latter of the harvest period. Earlier in the experiment there was a large ecological transient where variation in adult numbers was very high in the threshold adult harvest treatment. This is likely linked to the same very high harvest rates above 50% due to initial high population densities at the onset of harvesting. That our study includes environmental variation, and/or a model organism with complex life history more like that of many harvested species, might explain the difference in results from a previous experiment that suggested threshold harvesting methods are more conservative (Fryxell et al. 2005). The objective to reduce temporal variability in ecosystem service provision, as is a primary objective of threshold harvest management, has been called into question recently (Carpenter et al. 2015). Management to reduce short‐term variability in a fisheries model has unintended consequences that include greater extinction risks due to the interactions between environmental variance and management. Clearly from our own results, and taking this recent analysis into consideration, threshold harvest methods cannot be recommended as a more conservative harvesting strategy without further study.

## Conclusion

We have presented a microcosm study of invertebrates and provided experimental evidence to answer our two main questions; harvest induced age/stage/size‐truncation results in less stable populations and this is associated with significantly increased population variance only in variable environments. The likely mechanism behind this result is switching between additive and over compensatory changes in maturation, reproduction and survival during food limited and unlimited periods respectively (Cameron and Benton 2004). Such over‐compensatory mechanisms are known to cause populations to overshoot their carrying capacity at any point in time. Our study is conducted in a simplified environment where autocorrelated and random variance were considered separately. In nature, variation can occur over many time scales, with some components for example being high frequency (blue noise), random (white noise), and low frequency (red noise) occurring simultaneously. This study shows that the type of variation can make a very big difference to predictions of population responses to mortality, and this can be experimentally explored in model systems.

## Data Accessibility

The original time series and body size data from these experiments are available to download from DRYAD entry number http://dx.doi.org/10.5061/dryad.bq135.

## Conflict of Interest

None declared.

## Supporting information


**Figure S1.** Treatment time series of adult numbers predicted from General Additive Model.
**Figure S2**. Population time series averaged across six replicates of all harvest and environmental variation treatment combinations.
**Figure S3.** Plot of correlation structures between long term averages of adult versus juvenile population densities per replicate population tube.
**Figure S4**. Plot of centred moving average of population size and coefficient of variation of population size for 10 and 20 week windows.
**Table S1**. Summary of the effects of environmental variation and harvesting on the size of adult, juvenile egg or total soil mite populations.
**Table S2**. Summary of the effects of environmental variation and harvesting on the variance of adult, juvenile egg or total soil mite populations.
