# Supplementary material for: Harvested populations are more variable only in more variable environments
Source: Ecol Evol. 2016 May 24;6(12):4179–91. doi: 10.1002/ece3.2164 (PMC4884197; doi:10.1002/ece3.2164)
Supplement: Supplementary file 1 — Figure S1. Treatment time series of adult numbers predicted from General Additive Model. Figure S2. Population time series averaged across six replicates of all harvest and environmental variation treatment combinations. Figure S3. Plot of correlation structures between long term averages of adult versus juvenile population densities per replicate population tube. Figure S4. Plot of centred moving average of population size and coefficient of variation of population size for 10 and 20 week windows. Table S1. Summary of the effects of environmental variation and harvesting on the size of adult, juvenile egg or total soil mite populations. Table S2. Summary of the effects of environmental variation and harvesting on the variance of adult, juvenile egg or total soil mite populations. [file ECE3-6-4179-s001.docx]

Electronic Supplementary Material for

**Stage-structured harvesting interacts with environmental variability to determine population size and variance**

T.C. Cameron*, D. O’Sullivan, A. Reynolds, J. Hicks, S.B. Piertney, T.G. Benton

* To whom correspondence should be addressed.

TCC (tcameron@essex.ac.uk)

This PDF file includes:

Figure S1: Treatment time series of adult numbers predicted from General Additive Model

Figure S2: Population time series averaged across six replicates of all harvest and environmental variation treatment combinations.

Figure S3: Plot of correlation structures between long term averages of adult versus juvenile population densities per replicate population tube.

Figure S4: Plot of centred moving average of population size and coefficient of variation of population size for 10 and 20 week windows.

Table S1: Summary of the effects of environmental variation and harvesting on the size of adult, juvenile egg or total soil mite populations.

Table S2: Summary of the effects of environmental variation and harvesting on the variance of adult, juvenile egg or total soil mite populations.


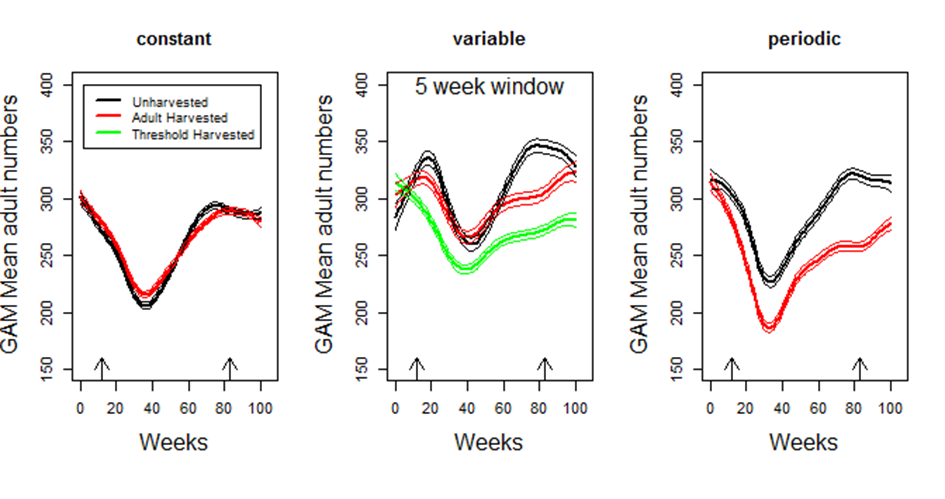


Constant Random Periodic

**Figure S1.** Each plot shows the fitted time series of mean ± 1s.e. adult stage abundance as predicted from a General Additive Model (GAM) fit to the CV of adult abundance over a centred moving 5 week window (i.e. generation time). Time series are shown for unharvested, proportional and threshold harvest populations in constant (left column), randomly variable (middle) and periodic food supply (right) environments. Degrees of freedom (DF) for the GAMs were chosen through model simplification, and determining the minimum DF that could best represent all CV time series within 5 centred week moving windows (i.e. 6 DF). Arrows show start and end of harvesting. Plots previously published in (Cameron et al., 2014).


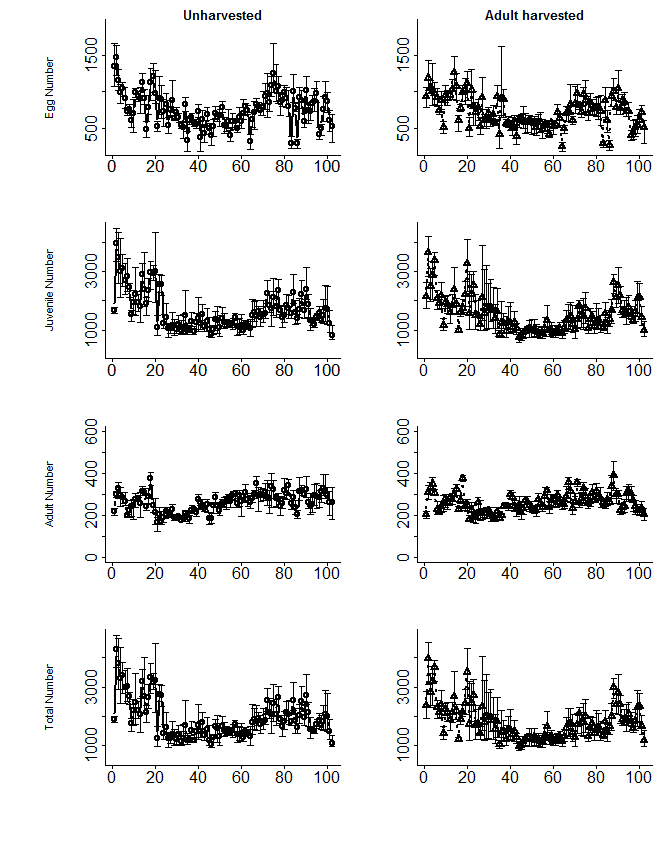


**A**

Figure S2. Mean stage specific and total population density per week of experiment in unharvested, Proportional Adult harvested (40% week during weeks 13-83) and Threshold Adult harvested (see methods) populations. Populations are fed the same average food daily either in **A.** Constant, **B.** Randomly variable or **C**. Periodically varying amounts (see methods). Continued on next pages.


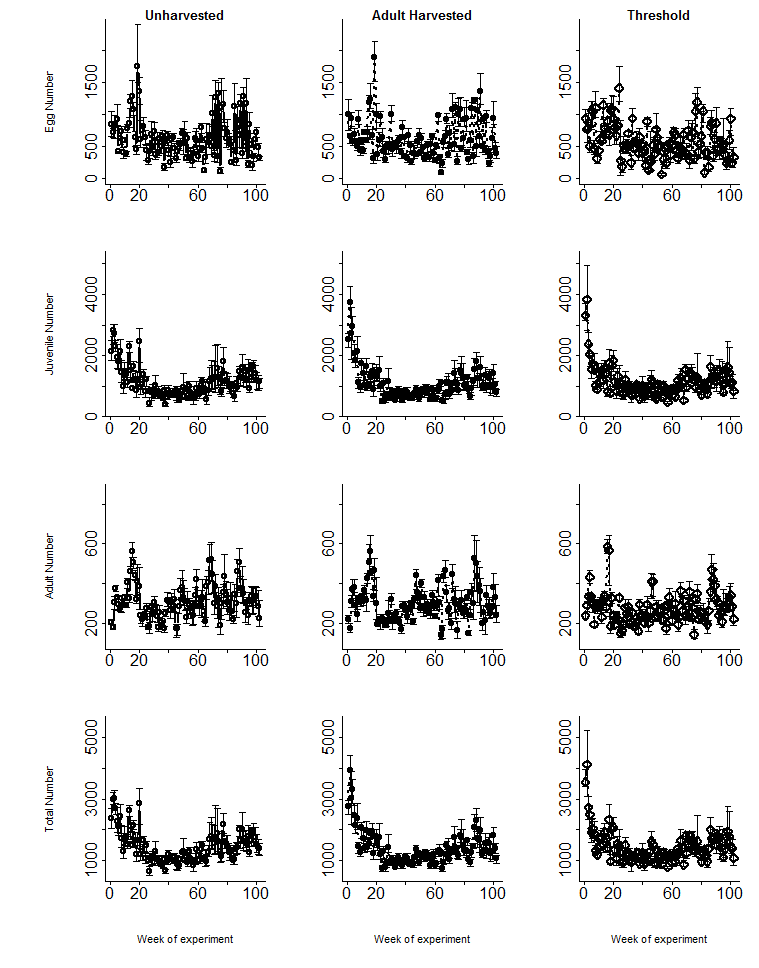


**B**

Continued from Figure S2A


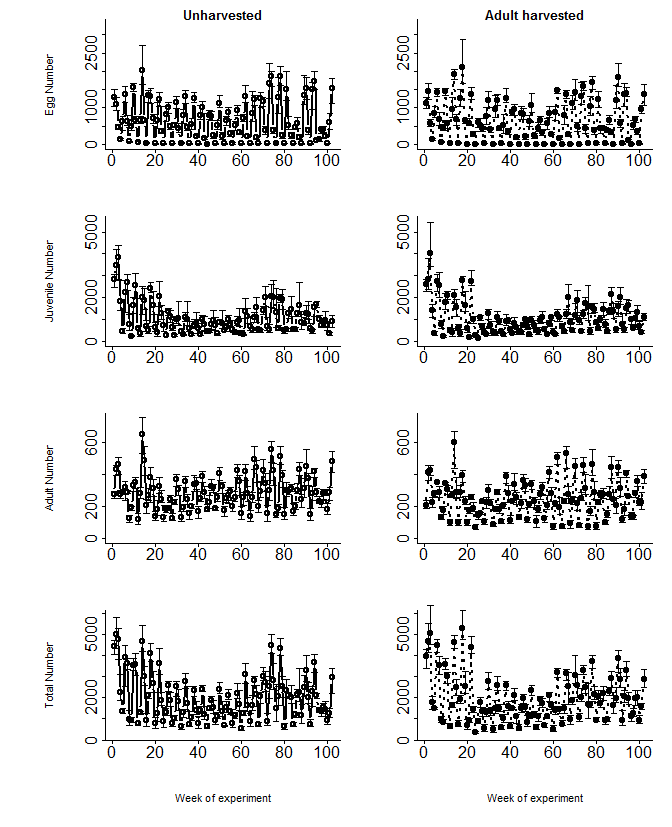


**C**

Continued from Figure S2B

Figure S3. Plot of linear correlation structures between long term averages of adult versus juvenile population densities per tube. Breakdown of explanatory power of this relationship occurs when moving from constant to variable environments as the adult and juvenile stage densities change differently in response to harvesting dependent on food availability.


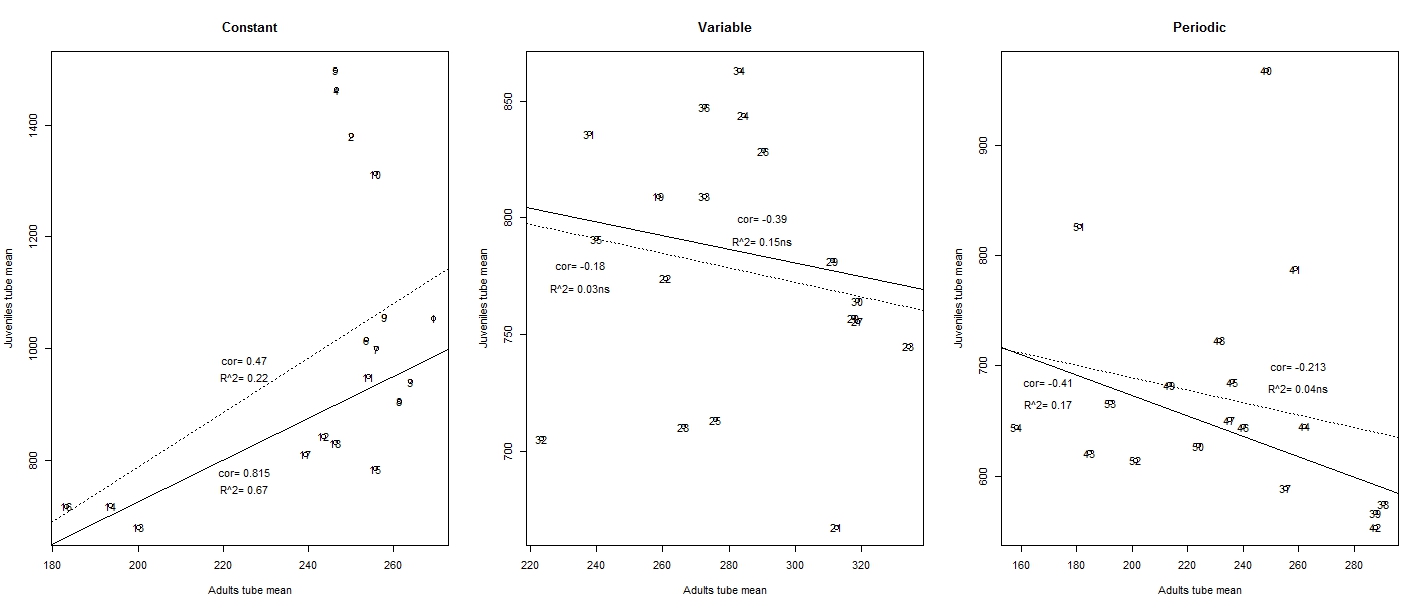


Constant Random Periodic

Constant Random Periodic


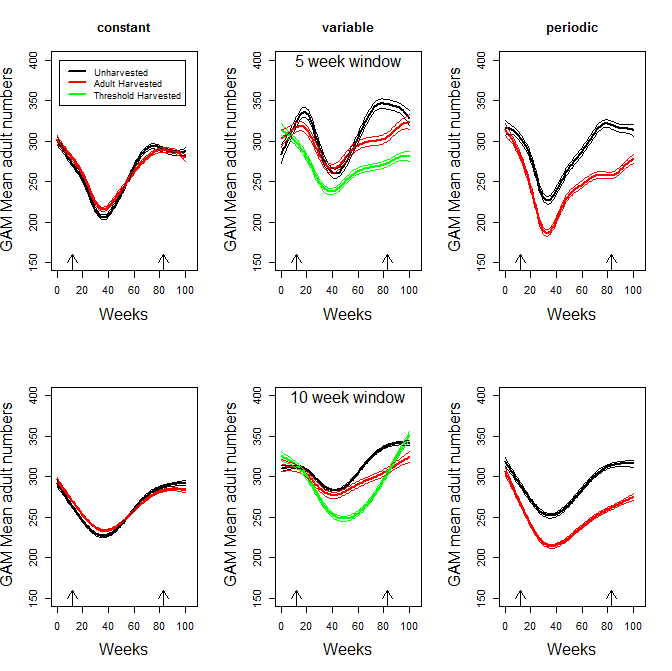


a


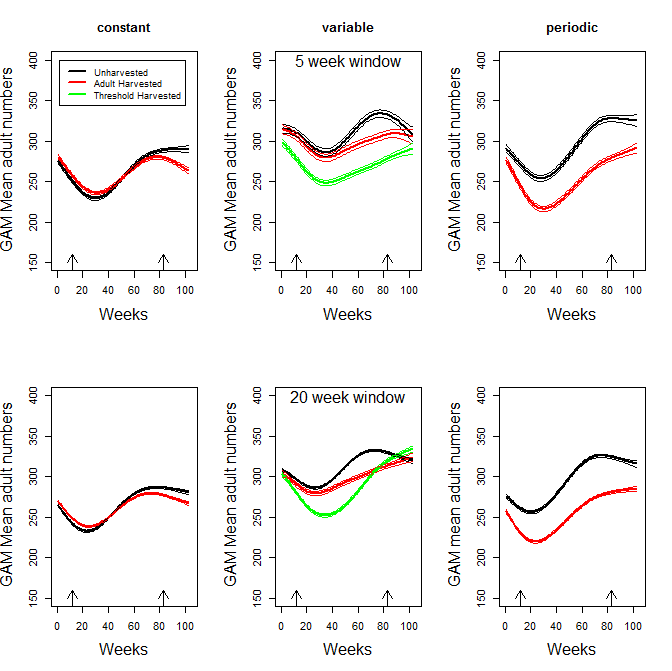

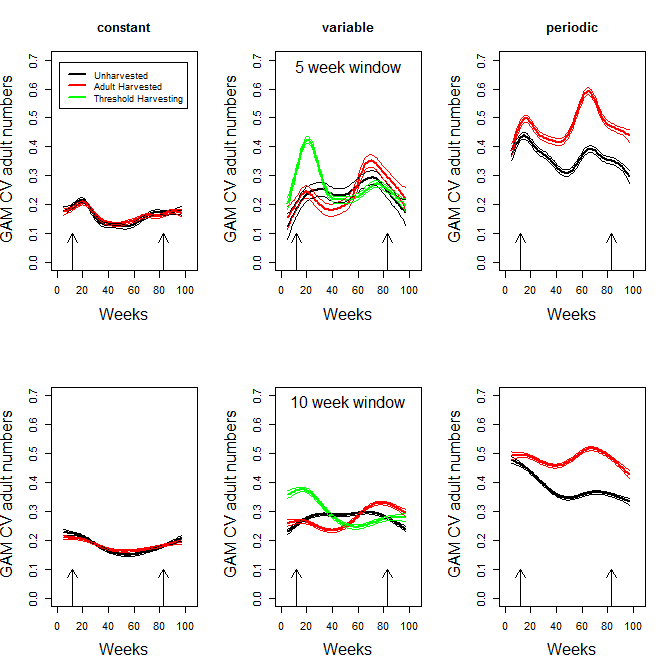

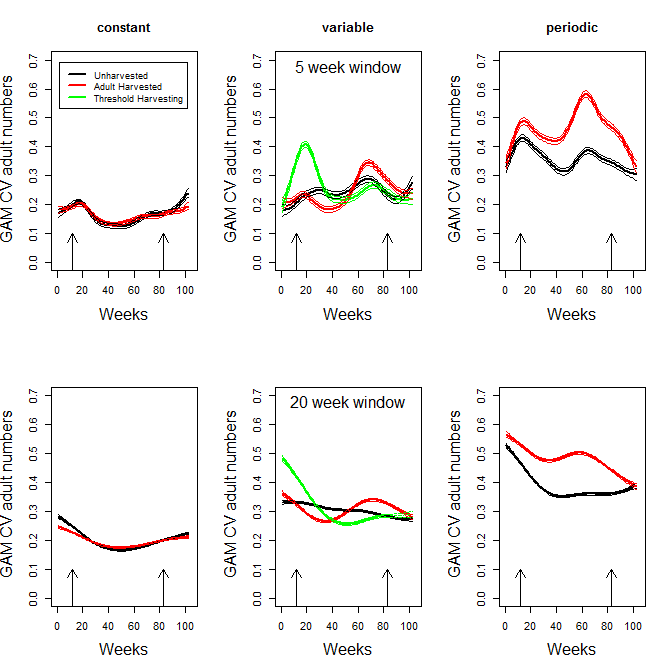


d

c

b

Figure S4. Each plot shows the fitted time series of mean ± 1s.e. of average population size (**a,b**) or coefficient of variation (CV) of adult stage abundance (**c,d**) as predicted from a General Additive Model (GAM) fit to the adult abundance over a centred moving 10 (**a,c**) or 20 week window (**b,d**). Time series are shown for unharvested (black), proportional (red) and threshold harvest (green) populations in constant (left column), randomly variable (middle) and periodic food supply (right) environments. See Analysis section in main manuscript for further details. Time series are qualitatively similar to 5 week window representing approximate generation time.

Table S1. The table summarises the effects of environmental variation and harvesting on the size of adult, juvenile egg or total soil mite populations. Comparisons are between time series from week 60-80. Percentages, (% in parentheses), are in reference to difference between variable and constant unharvested environments; or in the case of harvested populations the difference between the unharvested treatments within each environment. The difference is statistically significant as detailed in Table 1 and/or in the main text, unless otherwise stated by “ns”.

|  | Adults | Juveniles | Eggs | Total |
| --- | --- | --- | --- | --- |
| Constant | 290 | 1577 | 833 | 1868 |
|  |  |  |  |  |
| Random | 342 | 1100 | 651 | 1442 |
| % diff. from constant | (+17.7) | (-30.2) | (-21.8) | (-22.2) |
| Periodic | 311 | 1099 | 700 | 1411 |
| % diff. from constant | (+7.26)ns | (-30.3) | (-16.0) | (-24.4) |
| Constant Adult Harvest | 283 | 1259 | 720 | 1542 |
| % diff. from constant | (-2.6) ns | (-20.1) | (-13.6) | (-17.4) |
| Random Adult Harvest | 294 | 976 | 629 | 1270 |
| % diff. from random | (-14.0) | (-10.1) | (-3.4) ns | (-12.3) |
| Random Threshold Harvest | 262 | 1069 | 573 | 1332 |
| % diff. from random | (-23.1) | (-2.9)ns | (-11.9) | (-7.7) |
| Periodic Adult Harvest | 251 | 1062 | 687 | 1313 |
| % diff. from periodic | (-19.3) | (-3.4) ns | (-1.9) ns | (-6.9) ns |

Table S2. The table summarises the effects of environmental variation and harvesting on the variance of adult, juvenile egg or total soil mite populations (Coefficient of Variation). Comparisons are between time series from week 60-80. Percentages, (% in parentheses), are in reference to difference between variable and constant unharvested environments; or in the case of harvested populations the difference between the unharvested treatments within each environment. The difference is statistically significant as detailed in Table 1 and/or in the main text, unless otherwise stated by “ns”. “ms” = marginally significant P>0.1

|  | Adults | Juveniles | Eggs | Total |
| --- | --- | --- | --- | --- |
| Constant | 0.198 | 0.323 | 0.287 | 0.281 |
|  |  |  |  |  |
| Random | 0.351 | 0.439 | 0.625 | 0.341 |
| % diff. from constant | (77.7) | (35.8) | (117.6) | (21.4) |
| Periodic | 0.470 | 0.690 | 0.997 | 0.599 |
| % diff. from constant | (137.9) | (113.2) | (246.9) | (113.4) |
| Constant Adult Harvest | 0.172 | 0.371 | 0.267 | 0.310 |
| % diff. from constant | (-12.9) | (14.7) | (-6.9)ns | (10.5)ms |
| Random Adult Harvest | 0.345 | 0.404 | 0.537 | 0.303 |
| % diff. from random | (-1.8)ns | (-8.1)ns | (-14.1) | (-10.9) |
| Random Threshold Harvest | 0.270 | 0.354 | 0.515 | 0.251 |
| % diff. from random | (-22.9) | (-19.3) | (-17.6) | (-26.2) |
| Periodic Adult Harvest | 0.625 | 0.594 | 0.920 | 0.529 |
| % diff. from periodic | (32.9) | (-13.8) | (-7.7)ns | (-11.6) |

**Data Accessibility**

Time series of the stage-specific and total population sizes for each experimental treatment will be made available at publication using a suitable online database (i.e. either DRYAD or NERC Global Population Dynamics Database, possibly with 1 year embargo). Size measurements for published treatments will also be made available.

CAMERON, T. C., PLAISTOW, S. J., MUGABO, M., PIERTNEY, S. B. & BENTON, T. G. 2014. Eco-evolutionary dynamics: experiments in a model system. *Advances in Ecological Research,* 50.
